# Growth Analysis of Wheat Using Machine Vision: Opportunities and Challenges

**DOI:** 10.3390/s20226501

**Published:** 2020-11-14

**Authors:** Mohammad Ajlouni, Audrey Kruse, Jorge A. Condori-Apfata, Maria Valderrama Valencia, Chris Hoagland, Yang Yang, Mohsen Mohammadi

**Affiliations:** 1 Department of Agronomy, Purdue University, 915 West State Street, West Lafayette, IN 47907, USA; majl@just.edu.jo (M.A.); kruseas@purdue.edu (A.K.); jacondor@purdue.edu (J.A.C.-A.); choaglan@purdue.edu (C.H.); yang1527@purdue.edu (Y.Y.); 2 Department of Plant Production, Jordan University of Science and Technology, Ar Ramtha 3030, Jordan; 3 Departament Académico de Biología, Universidad Nacional de San Agustín de Arequipa, 117 Arequipa, Perú; mvalderramav@unsa.edu.pe

**Keywords:** machine vision, plant phenotyping, digital growth analysis, relative growth rate, wheat

## Abstract

Crop growth analysis is used for the assessment of crop yield potential and stress tolerance. Capturing continuous plant growth has been a goal since the early 20th century; however, this requires a large number of replicates and multiple destructive measurements. The use of machine vision techniques holds promise as a fast, reliable, and non-destructive method to analyze crop growth based on surrogates for plant traits and growth parameters. We used machine vision to infer plant size along with destructive measurements at multiple time points to analyze growth parameters of spring wheat genotypes. We measured side-projected area by machine vision and RGB imaging. Three traits, i.e., biomass (BIO), leaf dry weight (LDW), and leaf area (LA), were measured using low-throughput techniques. However, RGB imaging was used to produce side projected area (SPA) as the high throughput trait. Significant effects of time point and genotype on BIO, LDW, LA, and SPA were observed. SPA was a robust predictor of leaf area, leaf dry weight, and biomass. Relative growth rate estimated using SPA was a robust predictor of the relative growth rate measured using biomass and leaf dry weight. Large numbers of entries can be assessed by this method for genetic mapping projects to produce a continuous growth curve with fewer replicates.

## 1. Introduction

Crop growth analyses have been integral to crop physiology over the last century and entailed calculation of net assimilation rates [1,2]. Blackman [3] introduced the concept of accumulation of dry weight, later interpreted as rate of change in weight or relative growth rate (RGR). Blackman [3] also introduced the efficiency index of dry weight production and drafted the compound interest law as a simple equation that best describes the growth of annual plants:

W_1_ = W_0_ e^rt^ (1)

where W_1_ = the final weight, W_0_ = the initial weight, r = the efficiency index of dry weight production, t = time, and e is the base of natural logarithms. Since then, crop physiologists attempted to explain growth quantitatively using agricultural inputs such as fertilizer supply, environmental conditions, and periodic measurements of leaf area or dry weight as explanatory variables or responses at intervals throughout the plant growth from sowing to maturity.

Measuring traits for building growth models are destructive and demand multiple replications at short intervals because a trait measurement from a given plant cannot be repeated over time. To cope with such practical limitation, researchers measure multiple specimens from randomly selected plants, which still carry environmental and plant-to-plant variability. Another shortfall is that while growth is continuous, these models use measurements from growth “intervals” and do not use continuous measurements due to the low number of replicates. For example, leaf area index was modeled in corn using only three data points [4], or leaf area index and net assimilation rate in sunflower were reported using only one measurement at flowering [5]. Setiyono et al. [6] measured leaf area index in soybean using up to 12 field measurements. Absolute growth rate and leaf area duration in cowpea were reported by measurements from four sampling dates [7]. In summary, utilizing plant growth models requires a large number of replicates, and yet the intrinsic nature of destructive sampling imposes errors caused by environmental conditions pertinent to different samples.

In recent years, the plant community recognizes that the ability to produce massive phenotyping data is critical for deciphering genetics of complex traits and improving crops [8]. For example, when there is a need to screen a sufficiently large plant population for a quantitative trait analysis, e.g., grain size or abiotic stress tolerance, considering replicated trials over multiple locations makes phenotyping laborious and technically challenging. In some cases, the labor-intensive and costly nature of conventional field phenotyping led many crop breeding programs to make a single measurement of final yield for replicated plots in multiple environments [9,10,11]. However, yield itself is one of the most poorly inherited traits in crop breeding [12].

The “phenotyping bottleneck” described above is often due to the manual nature of trait measurements, e.g., a ruler, a weighing machine, and other available devices [13]. Using manual and destructive techniques, plant growth can be characterized by simple morphological measurements or crop growth parameters such as plant height, leaf area, leaf area index, and crop growth rate [14]. More recently, the face of plant phenotyping has changed to non-destructive methods based on advanced sensing technologies known as high-throughput phenotyping (HTP). Unlike traditional destructive sampling methods, HTP methods allow documentation of plant growth, biomass accumulation, and stress status under experimental conditions, e.g., water limitation, and advantageously, on the same plant over time (without destruction) on a continuous basis [15].

One approach to continuously and non-destructively evaluate growth is machine vision. Red Green Blue (RGB) imaging and image processing enable the inference of changes and growth of plant size and shape over time. For example, Yang et al. [16] analyzed high-throughput images to measure plant height, green leaf area, dry weight, and plant compactness. These methods lead to a significant increase in plant growth characteristics on a continuous basis with reduced replicates. HTP has been used for complex traits such as drought tolerance, growth rate response, and genomic regions linked to biomass in *Arabidopsis* [17], to describe floral development of wild *Antirrhinum majus* [18], to generate realistic rendering of trees in 3-D [19], and for applications in rust resistance phenotyping in wheat [20].

Wheat growth and development prior to heading can be regarded as the development of foliar tissue, i.e., leaves and culms that intercept solar radiation and then convert the atmospheric carbon to biomass [21]. Therefore, measurements of leaf area (LA) as the functional area of light interception and leaf dry weight (LDW) as one indicator of plant biomass are important. Measuring these traits using low-throughput methods involves destructive sampling and is time consuming. Because of the time and resource expensive nature of low-throughput methods, accurate, rapid, and nondestructive methods for plant measurements are needed. While previous studies such as that conducted by Campillo et al. [21] used imaging strategies for measuring leaf area, their work only used a single top-view image of tomato plants. Similarly, Easlon and Bloom [22], used a single image for estimation of leaf area in *Arabidopsis*.

The goal of this study was to demonstrate the use of machine vision and automated phenotyping by showing whether a high-throughput surrogate in wheat can accurately and rapidly estimate the growth and growth rate of wheat. The high-throughput image-based surrogate we chose to express growth traits was side projected area (SPA). One of the key steps in applying the RGB images for plant phenotyping is segmentation, which is to separate the targets of interest from the background [23]. Most of the current high-throughput plant phenotyping facilities leverage color index-based technologies, which establish segmentations by finding the difference in the color between the targets of interest and the background [24,25,26,27,28]. This method could be problematic, especially when the color in the target plants or background changes due to differences in species, varieties, or growth stage [29], or when shadows present or background scenes vary [30], making it challenging to adopt these techniques for high-throughput, automated image segmentation in the HTP facility data pipeline. In this study, we used correlative measurements to demonstrate that low-throughput traits measured in consecutive time points can be predicted with high accuracyby incorporating high throughput surrogate SPA using this method of segmentation.

## 2. Materials and Methods

### 2.1. Genotypes and Growing Condition

Plant materials were two spring wheat genotypes, Yecora Rojo and Seri-82, developed by the International Maize and Wheat Improvement Center (CIMMYT). Yecora-Rojo (pedigree: Ciano 67//Sonora 64/Klein Rendidor/3/II-8156 = II23584) is a released cultivar (CItr 17414) in California. Seri-82 (PI 591774) is a derivative of Kavkaz with wide geographical adaptation (pedigree: Kavkaz/Buho sib//Kalyansona/Bluebird). The potting medium was a 1:2 volumetric ratio of fine clay (Profile) to organic bagged soil (Sungro). Each pot was filled with approximately 2.2 ± 0.06 kg freshly prepared medium, which filled the pot all the way, leaving 3 cm of the pot empty. Our saturation optimization experiment revealed that the addition of 2.8 kg water will bring each pot to saturation (field capacity). This is a 126% gravimetric water content at saturation. For watering, we irrigated the plants using 500 mL (500/2800 = 18% of water content in saturated state) of 80 ppm nutrient solution of Peters Excel Cal Mg 15–5–15, six days per week. Initially, three seeds were planted, and upon successful emergence of one seedling, the others were extracted from the soil. Plants were grown in a walk-in controlled environment at 60% relative humidity (RH) under 800 µmol of photosynthetically active radiation (PAR), provided by an array of ceramic metal halide lamps (Philips Elite Agro T12), with a 14 h photoperiod, where the day and night temperatures were 25 and 20 °C, respectively. Plants were grown in the controlled environment growth chamber for a total of 53 days.

### 2.2. Experimental and Imaging Procedure

Each genotype was planted in 40 pots, keeping one single plant in each pot. The 40 pots were randomly divided into eight groups of five pots each. In each sampling time, i.e., 21, 25, 30, 35, 39, 44, 49, and 53 days after planting (DAP), five pots were imaged to produce high throughput surrogates and immediately destructively analyzed to produce ground-truth phenotype data. The plants were imaged in a closed imaging booth. At each imaging time points, plants were carried into the imaging booth on an automated conveyer belt and then stood on a rotation table, where we captured side view images at 12 angles, each rotating 30°. Plants were imaged using a custom-made RGB imager (Aris, Eindhoven, Netherlands), with a standard 5 megapixel CMOS sensor providing a 23 fps frame rate (Basler Ace, Ahrensburg, Germany). A monochrome camera (acA2440-20gm, Basler Ace, Ahrensburg, Germany) with a CMOS sensor of the same resolution and the same frame rate was equipped with a long-pass filter to capture chlorophyll fluorescence images. The imaging box was equipped with a blue light emitting diode (LED) array and a white LED array that provided the strobing light for image acquisition. Every time that a plant came into the imaging position on the rotation table, the blue LED array fired up to excite a chlorophyll fluorescence signal from the plant. The monochrome camera was synchronized with the blue LED light strobe for chlorophyll fluorescence image acquisition. The white LED then fired up, synchronized with the color camera to acquire an RGB image of the plant. The images that were taken by the two cameras were aligned by using a built-in image registration function.

### 2.3. Image Analysis

The image analysis processes started with creating the segmentation mask using the monochrome chlorophyll fluorescence image. Using the collected chlorophyll fluorescence image, Otsu’s [31] algorithm was adopted to establish a threshold to separate foreground and background based on the histogram of the gray values in the image. A binary mask was generated using this threshold; any pixels with values smaller than the threshold were considered background and assigned a value of zero (0); otherwise, the pixel belonging to the plant was assigned a value of one (1). A sequence of binary image morphological operations, including opening and closing operations, followed the binarization to minimize undesirable noises before establishing the final mask of the plant. Using the established mask, traits such as the side-projected plant area, the overall plant height, and width of the plant, etc., were calculated. The side projected area (SPA) of the plant was calculated as the sum of the pixels in the mask. The plant height was calculated as the length of the rectangle that enclosed the plant mask. Initially, these measurements were represented in number of pixels. A set of pre-established calibration functions then converted the pixel numbers into physical units (e.g., cm and cm^2^). All image analysis processes were implemented in MATLAB (Mathworks, Natick, MA, USA, Version 2019a). The average of SPA derived from 12 different angles was used to indicate each plant’s SPA. According to this, for each sampling time point and each variety, there were five replicates of SPA data. The five plants of each genotype were then destroyed for low-throughput trait analysis.

### 2.4. Trait Measurement

Destructive sampling began nearly two weeks after plant emergence when plants reached a height of 20 cm. In this experiment, eight sampling time points were completed, including 21, 25, 30, 35, 39, 44, 49, and 53 days after planting (DAP), where in each time point five pots from each genotype were destructively characterized for total leaf area and total phytomass production. Traits measured destructively were leaf area, leaf dry matter, total phytomass (above-ground dry matter), heading date, tiller number, and number of spikes per plants. Leaf area (LA) was measured using an LI-3100 Area Meter (LI-COR, inc. Lincoln, NE, USA) and expressed as cm^2^ plant^−1^ (or cm^2^). Plant tissues were dried at 65 °C for 72 h. The dry weight of plant leaves, leaf dry weight (LDW), was measured after drying and expressed in mg plant^−1^ (or mg). Above-ground biomass (BIO), representing all dry matter above-ground, was also measured after drying and expressed in mg plant^−1^ (or mg). Relative growth rate (RGR) was measured based on biomass produced and defined as total plant dry weight increase in a time interval in relation to the initial weight and expressed as gg^−1^ day^−1^ [3]. In addition, we calculated RGR based on the increases in unit time of the side projected area and expressed it as mm^2^ mm^−2^ day^−1^.

### 2.5. Data Analysis

For demonstrating growth across the time points and differential growth between the two genotypes, we used two-way analysis of variance (ANOVA) with time point and genotype as main factors, with consideration of the interaction effect of genotype and time point. This analysis was performed on three low throughput traits, namely BIO, LDW, and LA, as well as the high-throughput trait SPA. For demonstrating how well SPA predicts other low throughput traits, we used simple linear regression by taking SPA as an independent variable and BIO, LDW, and LA as dependent variables in each model. For each model, the coefficient of determination (R^2^) and the Pearson correlation coefficient (r) of SPA with each of the low throughput traits were reported. Two-way ANOVA was performed using “lm”, “anova”, and “summary” commands in R environment [32] with five replicates. All graphs were made using Microsoft Excel.

## 3. Results

### 3.1. Low Throughput Phenotyping (LTP)

The progression of LTP traits are shown in Table 1. In Yecora-Rojo, LA increased from 160 to 810 cm^2^ plant^−1^. In Seri-82, LA increased from 118 to 1800 cm^2^ plant^−1^. A similar increasing trend was observed in LDW, with Seri-82 always being greater than Yecora-Rojo beyond 30 DAP. The BIO showed an increasing trend as well, but Yecora-Rojo BIO was significantly greater than Seri-82 BIO. The total biomass of Yecora-Rojo at 21 DAP was 920 mg, rising to 45,390 mg at the eighth time point (53 DAP). In Seri-82, BIO increased from 730 mg to 41,600 mg over the same period.

**Figure 1:**
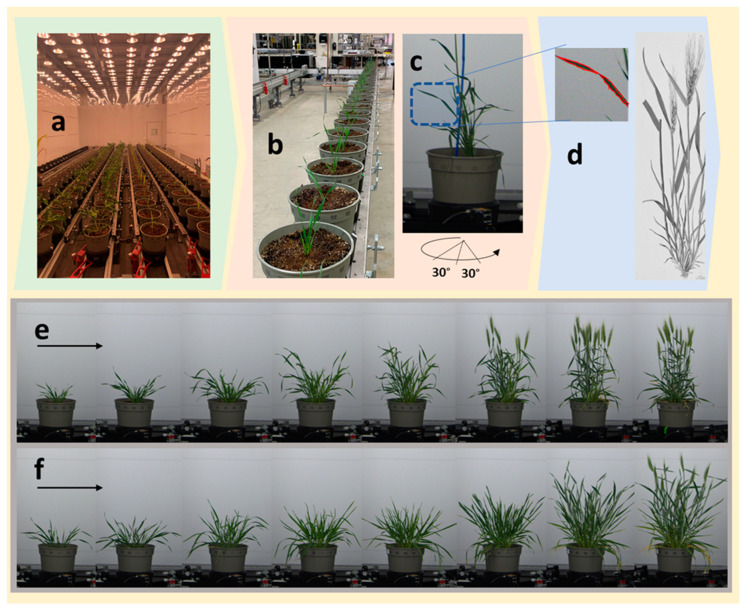
The phenotyping facility. The controlled growth environment (**a**), automated conveyer (**b**), the rotating station inside the imaging tower (**c**), and demonstration of detection of leaf edges used for deriving side projected area (SPA) (**d**) are shown. The lower panel shows the progression of growth from the first time point (left) to the eight time point (right), captured by RGB camera from representative Yecora-Rojo (**e**) and Seri-82 (**f**) genotypes.

The other difference among the two genotypes was noted in days of heading and number of tillers. Heading occurred for Yecora-Rojo at 39 DAP, but not until between 49 DAP and 53 DAP time points for Seri-82 (Figure 1). Yecora-Rojo produced a total of 25 tillers plant^−1^, of which 24 were fertile. Seri-82 produced 21 tillers, with only 8 fertile tillers at 53 DAP. The tillering stage of the two genotypes was also different by about 7 d.

The two-way ANOVA indicated that the individual effects of time point and genotype were highly significant on the four traits, indicating that the LA, LDW, BIO, and SPA increased in both genotypes continuously from the first time point to the last time point, and that the two genotypes showed different growth patterns (Table 2). The interaction effect of time point and genotype was only significant for LDW and LA.

### 3.2. The Predictive Performance of High Throughput Phenotyping

The SPA showed the same trend as the LTP traits (Table 1). It increased from 12,542 to 81,479 mm^2^ in Yecora-Rojo and from 10,882 to 105,419 mm^2^ in Seri-82 during the same period. Our objective was to examine how SPA predicts LTP traits. Therefore, we fitted linear regression by taking SPA as predictor variable and the LTP traits, i.e., LA, LDW, and BIO, as dependent variables. The highest prediction accuracy was observed for LDW (R^2^ = 0.98 for Yecora-Rojo and R^2^ = 0.98 for Seri-82) followed by LA (R^2^ = 0.89 for Yecora-Rojo and R^2^ = 0.98 for Seri-82). BIO was linearly predicted with the least accuracy (R^2^ = 0.86 for Yecora-Rojo and R^2^ = 0.91 for Seri-82). While the relationship between SPA and LA (and LDW) was shown to be a linear fit, the relationship between SPA and BIO was not linear and resembled a quadratic or exponential trend. When SPA was used to predict LA, LDW, and BIO, the regression was highly significant. The correlation coefficient between SPA and LA, LDW, and BIO was found to be between 0.92 and 0.99 for Yecora-Rojo and between 0.91 and 0.99 for Seri-82 (Table 3).

### 3.3. Relative Growth Rate Measurements on the Basis of Low- and High-Throughput Traits

**Figure 2:**
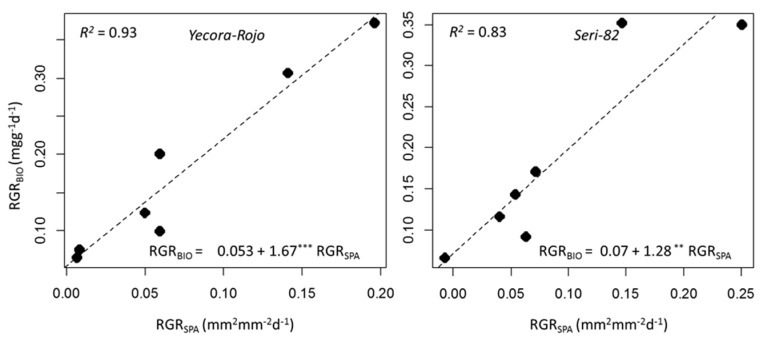
Simple linear regression of relative growth rate of biomass (RGR_BIO_) as predicted by relative growth rate derived from image-based surface projected area (RGR_SPA_) of Yecora-Rojo (**left**) and Seri-82 (**right**). The regression equations, significance levels (*** for 0.001 and ** for 0.01), and coefficient of determinations (R^2^) are shown on each figure. Because our experiment included eight time points, we could calculate RGR measurements for seven intervals.

Relative growth rate (RGR) is the increase of plant biomass relative to size. This parameter can be expressed for other traits such as LA, or any other quantitative traits that are subject to change over time. In our experiment, we measured relative growth rate for LDW, BIO, and SPA. The RGR values were relatively higher during 21–30 DAP, dropping to lower values over time (Table 4). For example, in Yacora-Rojo, RGR_LDW_ was 309.0 mgg^−1^day^−1^ during 21–25 DAP, dropping to 19.4 mgg^−1^day^−1^ during 49–53 DAP. The same figures for Seri-82 were 345.5 mgg^−1^day^−1^ during the 21–25 DAP interval and dropping to 4.8 mgg^−1^day^−1^ during the 49–53 DAP interval. The RGR_BIO_ in Yecora-Rojo decreased from 372.3 to 64.6 mgg^−1^day^−1^ and decreased from 349.3 to 65.3 mgg^−1^day^−1^ in Seri-82. Interestingly and with high accuracy, the RGR that was calculated based on SPA (RGR_SPA_) values also followed the same trend for both genotypes. It decreased from the first interval through to the last interval (Table 4). One objective of our study was to determine whether RGR measured on the basis of HTP can replace the time and large sample consuming LTP based measurements. The results showed that the regression relationship was significant, and that RGR_SPA_ could explain up to 93% of the variations observed in RGR_BIO_ (Figure 2). The coefficient of correlation of RGR_BIO_ and RGR_SPA_ was 0.97 for Yecora-Rojo and 0.91 for Seri-82, respectively.

### 3.4. Side-Projection Area Efficiency during Growth

**Figure 3:**
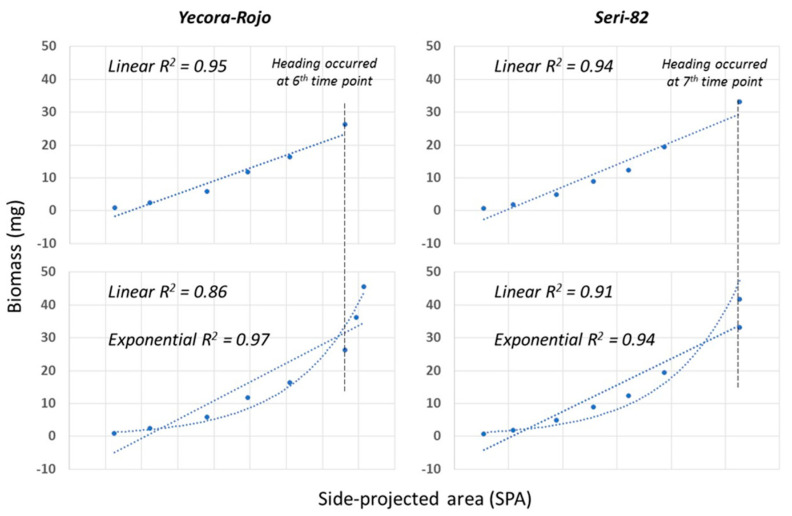
Simple linear regression of side-projected area (SPA) and biomass for the two genotypes.

Side-projected area is an efficient predictor of biomass up until heading (Figure 3, top panel). However, with the transitioning from vegetative growth to reproductive and emergence of heads, a linear function of SPA is a poor predictor of biomass, as indicated by a drop in the coefficient of determination (Figure 3, bottom panel), and underestimates the biomass. An exponentially fitted function provides a better prediction of biomass, as indicated by higher coefficients of determination.

## 4. Discussion

The overall goal of this study was to validate the manual and destructive growth analysis of wheat using non-destructive machine vision surrogates. Explaining crop growth by using high throughput phenotyping facilitates crop physiology measurements and accelerates crop genetics research for growth-related traits. In this study, machine vision supported by automated plant RGB imaging was used to produce surrogates for wheat growth with high accuracy. The accuracy of digital growth analysis was demonstrated by correlation between HTP and LTP ground-truth measurements. The correlation results reported in this study were also reported by previous researchers. Rajendran et al. [33] reported a positive relationship between image-based surrogates and shoot biomass (R^2^ = 0.94) and leaf area width (R^2^ = 0.92) in *Triticum monococcum* species, and Ahmad et al. [34] reported high correlations between manual and digital methods on leaf area index estimation for wheat (R^2^ = 0.53–0.93), barley (R^2^ = 0.58–0.96), and oat (R^2^ = 0.72–0.97). Similar results were obtained from other studies for wheat and barley [35]; soybean and corn [36]; *Arabidopsis* rosettes [22]; and forest tree seedlings [37].

Additionally, our results indicated that HTP can reduce the number of replicates and the magnitude of experimental error generated by plant-to-plant variation. For example, destructive sampling for eight time points and five replicates per time point requires 40 plants and yet may not be able to depict the changes in the rate of growth because of low resolution. In contrast, non-destructive image-based growth analysis can rely on fewer pots (e.g., five) and can provide a daily analysis of growth. Therefore, the overall benefits will be the time and cost that are needed for completing the growth rate measurements. This is significant for enabling assessment of large germplasm for crop improvement [38,39].

High throughput phenotyping can offer non-destructive methods that enable measurements on the same plant over time without the need to measure the plant destructively and thereby making it possible to track the growth and development of the plant over time or effects on growth responses in different environmental conditions. By establishing a high-dimensional data matrix that characterizes the plant’s growth and development, HTP enables in-depth analysis of the interactions among distinct traits, which provides opportunities to identify key parameters that correlate with desirable traits [40], such as drought tolerance. More importantly, the machine vision can eliminate confounding effects of plant-to-plant variation in repeated measurements of growth analysis. The ability to control the measurements of individual plants with higher accuracy measurements can open the opportunity for measurements of short lived mortal genetic mapping population (such as F2 or backcross mapping populations), where each individual of the population has a unique genetic identity. Another benefit is that HTP enables us to fill the existing gap in plant physiology by decreasing the interval between two consecutive measurements, where a trait measurement from a given plant can be repeated over a very short time, mimicking continuous measurement for continuous growth. This will enable the capture of small changes and their timing in plant growth.

Despite these advantages, we identified limitations in using HTP for digital growth analysis. One area of improvement for surrogate-based modeling of biomass is the time of transitioning from the vegetative to reproductive stage. After heading, SPA was not predictive of organ biomass and underestimated the biomass if modelled linearly, because spikes have higher specific weight than leaves and stems as they progress through the grain-fill process. Another area that needs to be approached cautiously is interpretation of results obtained from pot and single-plant measurements to field-scale analysis, because the true grain yield expression is always measured in the plant community in field and in direct interaction with the environment. Using single plant analysis clearly misses the effect of plant–plant interactions and competition.

## 5. Conclusions

In this study, we presented a method for accurate estimation of plant LA, LDW, and BIO using an HTP surrogate. We converted images taken from plants into numeric values that represented growth in each time point. This approach provided an accurate and practical model for the estimation of wheat leaf traits and shoot biomass as a substitute for the conventional destructive method with high accuracy. The results indicated that the method can reduce the number of replicates and the error generated by plant-to-plant variation with very short interval times compared to the destructive method. In this study, we calculated RGR using a low throughput method based on LA (RGR_LA_), LDW (RGR_LDW_), and BIO (RGR_BIO_) and showed that RGR based on the HTP surrogate (RGR_SPA_) is well correlated with those measured by LTP. HTP has shown to be promising for monitoring the growth and development of the plant over time and for studying plant responses to environmental stresses. However, methodological improvements are needed to better model the plant growth parameter to capture biomass changes after transitioning from the vegetative to reproductive stage.

## Figures and Tables

**Table 1:** Changes in leaf area, dry weight, biomass, and side-projected area across the eight time points for Yecora-Rojo and Seri-82 genotypes. Values are presented as mean ± SE (n = 5).

Measurements	21 DAP	25 DAP	30 DAP	35 DAP	39 DAP	44 DAP	49 DAP	53 DAP
**Yecora-Rojo**								
Leaf area (cm^2^)	160 ± 17	367 ± 55	601 ± 34	649 ± 28	726 ± 49	756 ± 31	771 ± 31	810 ± 30
Leaf dry weight (mg)	720 ± 70	1610 ± 280	3010 ± 120	3630 ± 110	4090 ± 240	4930 ± 100	5280 ± 90	5690 ± 90
Biomass (mg)	920 ± 090	2290 ± 460	5800 ± 460	11,620 ± 390	16,220 ± 980	26,220 ± 740	36,070 ± 1540	45,390 ± 910
Side projected area (mm^2^)	12,542 ± 960	22,359 ± 2463	38,115 ± 2198	49,408 ± 1175	61,093 ± 2732	76,230 ± 2238	79,450 ± 1476	81,479 ± 2137
**Seri-82**								
Leaf area (cm^2^)	118 ± 13	290 ± 33	572 ± 34	884 ± 45	1173 ± 85	1594 ± 158	1887 ± 77	1800 ± 105
Leaf dry weight (mg)	550 ± 70	1300 ± 150	3510 ± 400	5000 ± 300	7080 ± 350	10,000 ± 800	11,240 ± 520	11,450 ± 650
Biomass (mg)	730 ± 100	1800 ± 200	5000 ± 400	9000 ± 500	12,000 ± 190	19,000 ± 1000	32,980 ± 1030	41,600 ± 2310
Side projected area (mm^2^)	10,882 ± 1006	21,795 ± 1752	37,825 ± 1526	51,398 ± 2765	64,438 ± 4029	77,526 ± 4491	107,159 ± 1534	105,419 ± 2887

DAP denotes days after planting.

**Table 2:** Significance levels of time points, genotype, and their interaction for the four traits evaluated via two-way analysis of variance.

Source	df	Biomass	Leaf Dry Weight	Leaf Area	Side Proj. Area
Time point	7	<2.2 × 10^−16^ ***	<2.2 × 10^−16^ ***	<2.2 × 10^−16^	<2.2 × 10^−16^
Genotype	1	8.122 × 10^−5^ ***	1.949 × 10^−15^ ***	8.159 × 10^−16^ ***	0.02625 *
Genotype × Time point	7	0.1372	8.599 × 10^−10^ ***	1.179 × 10^−12^ ***	0.11584
R^2^		0.9357	0.9189	0.9137	0.921

Footnote: * and *** indicate significance at 0.05 and 0.001, respectively.

**Table 3:** Simple linear regression of leaf area (LA), leaf dry weight (LDW), and biomass (BIO) as predicted by side projected area (SPA). All regression models are significant (*p* < 0.01). R^2^ is the coefficient of determination, and r is the Pearson coefficient of regression.

Trait	Yecora-Rojo	R^2^	r	Seri-82	R^2^	r
LA	0.008 SPA + 178.85	88.9%	0.94	0.0188 SPA − 81.859	98.0%	0.99
LDW	0.066 SPA + 149.1	98.4%	0.99	0.1187 SPA − 782.3	97.9%	0.99
BIO	0.5709 SPA − 11,956	85.6%	0.93	0.3982 SPA − 8430.7	91.2%	0.95

**Table 4:** Changes of relative growth rate and net assimilation rate across intervals for the two genotypes. Values are presented as mean (n = 5).

Growth Parameter				DAP			
	21–25	25–30	30–35	35–39	39–44	44–49	49–53
**Yecora-Rojo**							
RGR_LDW_ (mgg^−1^ d^−1^)	309	173.9	41.2	31.7	41.1	14.2	19.4
RGR_BIO_ (mgg^−1^ d^−1^)	372.3	306.6	200.7	99	123.3	75.1	64.6
RGR_SPA_ (mm^2^ mm^−2^ d^−1^)	0.196	0.141	0.059	0.059	0.05	0.008	0.006
**Seri-82**							
RGR_LDW_ (mgg^−1^ d^−1^)	345.5	335.9	106	79.6	76	29.9	4.8
RGR_BIO_ (mgg^−1^ d^−1^)	349.3	352	170.2	90.9	115.7	142.8	65.3
RGR_SPA_ (mm^2^ mm^−2^ d^−1^)	0.251	0.147	0.072	0.063	0.041	0.054	−0.007

DAP: days after planting.
